# Supplementary material for: Are Women with Normal-Weight Obesity at Higher Risk for Cardiometabolic Disorders?
Source: Biomedicines. 2023 Jan 25;11(2):341. doi: 10.3390/biomedicines11020341 (PMC9953250; doi:10.3390/biomedicines11020341)
Supplement: Supplementary file 1 [file biomedicines-11-00341-s001.zip › biomedicines-2133087-supplementary.pdf]

		PBF	FBS	Cholesterol	TG	HDL	LDL	LDL / HDL	Cholesterol / HDL	VLDL	AST	ALT	ALP	insulin	HOMA_IR	HOMA_S_percentage	HOMA_B_percentage	Hs-CRP
PBF	R	1	.240**	.247**	.299**	-.559**	.376**	.521**	.503**	.299**	0.050	0.134	0.121	.193*	.201*	-0.146	0.048	.518**
	P-value		0.003	0.002	0.000	0.000	0.000	0.000	0.000	0.000	0.537	0.098	0.136	0.017	0.012	0.071	0.553	0.000
FBS	R	.240**	1	0.048	.320**	-0.089	-0.001	0.048	0.087	.320**	-0.029	0.019	0.064	0.111	.160*	-0.094	-.450**	.253**
	P-value	0.003		0.555	0.000	0.274	0.995	0.554	0.283	0.000	0.720	0.810	0.428	0.172	0.047	0.248	0.000	0.002
Cholesterol	R	.247**	0.048	1	.440**	-0.152	.888**	.704**	.710**	.440**	0.064	0.115	0.112	.172*	.177*	-0.107	0.071	0.149
	P-value	0.002	0.555		0.000	0.060	0.000	0.000	0.000	0.000	0.433	0.156	0.166	0.033	0.028	0.185	0.380	0.065
TG	R	.299**	.320**	.440**	1	-.379**	.276**	.412**	.549**	1.000**	0.012	0.128	0.057	0.095	0.112	-0.006	-0.113	.205*
	P-value	0.000	0.000	0.000		0.000	0.001	0.000	0.000	0.000	0.880	0.113	0.482	0.243	0.168	0.941	0.161	0.011
HDL	R	-.559**	-0.089	-0.152	-.379**	1	-.323**	-.703**	-.748**	-.379**	-0.023	-0.150	-0.158	-.169*	-.172*	.176*	-0.112	-.299**
	P-value	0.000	0.274	0.060	0.000		0.000	0.000	0.000	0.000	0.779	0.063	0.050	0.036	0.032	0.029	0.168	0.000
LDL	R	.376**	-0.001	.888**	.276**	-.323**	1	.861**	.747**	.276**	0.020	0.061	0.122	.200*	.202*	-0.155	0.122	.250**
	P-value	0.000	0.995	0.000	0.001	0.000		0.000	0.000	0.001	0.809	0.450	0.131	0.013	0.012	0.054	0.130	0.002
LDL / HDL	R	.521**	0.048	.704**	.412**	-.703**	.861**	1	.948**	.412**	0.025	0.112	0.137	.261**	.263**	-.200*	.167*	.328**
	P-value	0.000	0.554	0.000	0.000	0.000	0.000		0.000	0.000	0.757	0.166	0.090	0.001	0.001	0.013	0.038	0.000
Cholesterol / HDL	R	.503**	0.087	.710**	.549**	-.748**	.747**	.948**	1	.549**	0.061	.166*	0.147	.252**	.256**	-.183*	0.148	.290**
	P-value	0.000	0.283	0.000	0.000	0.000	0.000	0.000		0.000	0.455	0.039	0.069	0.002	0.001	0.023	0.068	0.000
VLDL	R	.299**	.320**	.440**	1.000**	-.379**	.276**	.412**	.549**	1	0.012	0.128	0.057	0.095	0.112	-0.006	-0.113	.205*
	P-value	0.000	0.000	0.000	0.000	0.000	0.001	0.000	0.000		0.880	0.113	0.482	0.243	0.168	0.941	0.161	0.011
AST	R	0.050	-0.029	0.064	0.012	-0.023	0.020	0.025	0.061	0.012	1	.722**	-0.002	0.052	0.049	-0.054	0.105	0.058
	P-value	0.537	0.720	0.433	0.880	0.779	0.809	0.757	0.455	0.880		0.000	0.978	0.519	0.548	0.510	0.195	0.473
ALT	R	0.134	0.019	0.115	0.128	-0.150	0.061	0.112	.166*	0.128	.722**	1	0.157	0.018	0.016	-0.050	0.056	0.133
	P-value	0.098	0.810	0.156	0.113	0.063	0.450	0.166	0.039	0.113	0.000		0.052	0.828	0.845	0.538	0.490	0.100
ALP	R	0.121	0.064	0.112	0.057	-0.158	0.122	0.137	0.147	0.057	-0.002	0.157	1	0.021	0.023	-0.101	0.022	0.113
	P-value	0.136	0.428	0.166	0.482	0.050	0.131	0.090	0.069	0.482	0.978	0.052		0.794	0.777	0.211	0.784	0.165
insulin	R	.193*	0.111	.172*	0.095	-.169*	.200*	.261**	.252**	0.095	0.052	0.018	0.021	1	.998**	-.796**	.801**	.217**
	P-value	0.017	0.172	0.033	0.243	0.036	0.013	0.001	0.002	0.243	0.519	0.828	0.794		0.000	0.000	0.000	0.007
HOMA_IR	R	.201*	.160*	.177*	0.112	-.172*	.202*	.263**	.256**	0.112	0.049	0.016	0.023	.998**	1	-.800**	.768**	.227**
	P-value	0.012	0.047	0.028	0.168	0.032	0.012	0.001	0.001	0.168	0.548	0.845	0.777	0.000		0.000	0.000	0.005
HOMA_S_percentage	R	-0.146	-0.094	-0.107	-0.006	.176*	-0.155	-.200*	-.183*	-0.006	-0.054	-0.050	-0.101	-.796**	-.800**	1	-.705**	-.169*
	P-value	0.071	0.248	0.185	0.941	0.029	0.054	0.013	0.023	0.941	0.510	0.538	0.211	0.000	0.000		0.000	0.036
HOMA_B_percentage	R	0.048	-.450**	0.071	-0.113	-0.112	0.122	.167*	0.148	-0.113	0.105	0.056	0.022	.801**	.768**	-.705**	1	0.038
	P-value	0.553	0.000	0.380	0.161	0.168	0.130	0.038	0.068	0.161	0.195	0.490	0.784	0.000	0.000	0.000		0.641
hsCRP	R	.518**	.253**	0.149	.205*	-.299**	.250**	.328**	.290**	.205*	0.058	0.133	0.113	.217**	.227**	-.169*	0.038	1
	P-value	0.000	0.002	0.065	0.011	0.000	0.002	0.000	0.000	0.011	0.473	0.100	0.165	0.007	0.005	0.036	0.641	

**. Correlation is significant at the 0.01 level (2-tailed).
*. Correlation is significant at the 0.05 level (2-tailed).

Supplementary table S1. Correlation between BFP with cardiometabolic markers.

Correlations

		WC	FBS	Cholesterol	TG	HDL	LDL	LDL / HDL	Cholesterol / HDL	VLDL	AST	ALT	ALP	Insulin	HOMA_IR	HOMA_S_P-valueercentage	HOMA_B_P-valueercentage	Hs-CRP
WC	R	1	.214**	.253**	.313**	-.511**	.359**	.502**	.492**	.313**	0.031	0.119	0.123	.178*	.188*	-0.126	0.039	.550**
	P-value		0.008	0.002	0.000	0.000	0.000	0.000	0.000	0.000	0.701	0.143	0.129	0.027	0.020	0.121	0.633	0.000
FBS	R	.214**	1	0.048	.320**	-0.089	-0.001	0.048	0.087	.320**	-0.029	0.019	0.064	0.111	.160*	-0.094	-.450**	.253**
	P-value	0.008		0.555	0.000	0.274	0.995	0.554	0.283	0.000	0.720	0.810	0.428	0.172	0.047	0.248	0.000	0.002
Cholesterol	R	.253**	0.048	1	.440**	-0.152	.888**	.704**	.710**	.440**	0.064	0.115	0.112	.172*	.177*	-0.107	0.071	0.149
	P-value	0.002	0.555		0.000	0.060	0.000	0.000	0.000	0.000	0.433	0.156	0.166	0.033	0.028	0.185	0.380	0.065
TG	R	.313**	.320**	.440**	1	-.379**	.276**	.412**	.549**	1.000**	0.012	0.128	0.057	0.095	0.112	-0.006	-0.113	.205*
	P-value	0.000	0.000	0.000		0.000	0.001	0.000	0.000	0.000	0.880	0.113	0.482	0.243	0.168	0.941	0.161	0.011
HDL	R	-.511**	-0.089	-0.152	-.379**	1	-.323**	-.703**	-.748**	-.379**	-0.023	-0.150	-0.158	-.169*	-.172*	.176*	-0.112	-.299**
	P-value	0.000	0.274	0.060	0.000		0.000	0.000	0.000	0.000	0.779	0.063	0.050	0.036	0.032	0.029	0.168	0.000
LDL	R	.359**	-0.001	.888**	.276**	-.323**	1	.861**	.747**	.276**	0.020	0.061	0.122	.200*	.202*	-0.155	0.122	.250**
	P- value	0.000	0.995	0.000	0.001	0.000		0.000	0.000	0.001	0.809	0.450	0.131	0.013	0.012	0.054	0.130	0.002
LDL / HDL	R	.502**	0.048	.704**	.412**	-.703**	.861**	1	.948**	.412**	0.025	0.112	0.137	.261**	.263**	-.200*	.167*	.328**
	P-value	0.000	0.554	0.000	0.000	0.000	0.000		0.000	0.000	0.757	0.166	0.090	0.001	0.001	0.013	0.038	0.000
Cholesterol / HDL	R	.492**	0.087	.710**	.549**	-.748**	.747**	.948**	1	.549**	0.061	.166*	0.147	.252**	.256**	-.183*	0.148	.290**
	P-value	0.000	0.283	0.000	0.000	0.000	0.000	0.000		0.000	0.455	0.039	0.069	0.002	0.001	0.023	0.068	0.000
VLDL	R	.313**	.320**	.440**	1.000**	-.379**	.276**	.412**	.549**	1	0.012	0.128	0.057	0.095	0.112	-0.006	-0.113	.205*
	P-value	0.000	0.000	0.000	0.000	0.000	0.001	0.000	0.000		0.880	0.113	0.482	0.243	0.168	0.941	0.161	0.011
AST	R	0.031	-0.029	0.064	0.012	-0.023	0.020	0.025	0.061	0.012	1	.722**	-0.002	0.052	0.049	-0.054	0.105	0.058
	P-value	0.701	0.720	0.433	0.880	0.779	0.809	0.757	0.455	0.880		0.000	0.978	0.519	0.548	0.510	0.195	0.473
ALT	R	0.119	0.019	0.115	0.128	-0.150	0.061	0.112	.166*	0.128	.722**	1	0.157	0.018	0.016	-0.050	0.056	0.133
	P-value	0.143	0.810	0.156	0.113	0.063	0.450	0.166	0.039	0.113	0.000		0.052	0.828	0.845	0.538	0.490	0.100
ALP	R	0.123	0.064	0.112	0.057	-0.158	0.122	0.137	0.147	0.057	-0.002	0.157	1	0.021	0.023	-0.101	0.022	0.113
	P-value	0.129	0.428	0.166	0.482	0.050	0.131	0.090	0.069	0.482	0.978	0.052		0.794	0.777	0.211	0.784	0.165
Insulin	R	.178*	0.111	.172*	0.095	-.169*	.200*	.261**	.252**	0.095	0.052	0.018	0.021	1	.998**	-.796**	.801**	.217**
	P-value	0.027	0.172	0.033	0.243	0.036	0.013	0.001	0.002	0.243	0.519	0.828	0.794		0.000	0.000	0.000	0.007
HOMA_IR	R	.188*	.160*	.177*	0.112	-.172*	.202*	.263**	.256**	0.112	0.049	0.016	0.023	.998**	1	-.800**	.768**	.227**
	P-value	0.020	0.047	0.028	0.168	0.032	0.012	0.001	0.001	0.168	0.548	0.845	0.777	0.000		0.000	0.000	0.005
HOMA_S_P-valueercentage	R	-0.126	-0.094	-0.107	-0.006	.176*	-0.155	-.200*	-.183*	-0.006	-0.054	-0.050	-0.101	-.796**	-.800**	1	-.705**	-.169*
	P-value	0.121	0.248	0.185	0.941	0.029	0.054	0.013	0.023	0.941	0.510	0.538	0.211	0.000	0.000		0.000	0.036
HOMA_B_P-valueercentage	R	0.039	-.450**	0.071	-0.113	-0.112	0.122	.167*	0.148	-0.113	0.105	0.056	0.022	.801**	.768**	-.705**	1	0.038
	P-value	0.633	0.000	0.380	0.161	0.168	0.130	0.038	0.068	0.161	0.195	0.490	0.784	0.000	0.000	0.000		0.641
Hs-CRP	R	.550**	.253**	0.149	.205*	-.299**	.250**	.328**	.290**	.205*	0.058	0.133	0.113	.217**	.227**	-.169*	0.038	1
	P-value	0.000	0.002	0.065	0.011	0.000	0.002	0.000	0.000	0.011	0.473	0.100	0.165	0.007	0.005	0.036	0.641	

**. Correlation is significant at the 0.01 level (2-tailed).
*. Correlation is significant at the 0.05 level (2-tailed).

Supplementary table S2. Correlation between WC with cardiometabolic markers.
